# Factors Affecting Attendance at and Timing of Formal Antenatal Care: Results from a Qualitative Study in Madang, Papua New Guinea

**DOI:** 10.1371/journal.pone.0093025

**Published:** 2014-05-19

**Authors:** Erin V. W. Andrew, Christopher Pell, Angeline Angwin, Alma Auwun, Job Daniels, Ivo Mueller, Suparat Phuanukoonnon, Robert Pool

**Affiliations:** 1 Barcelona Centre for International Health Research (CRESIB), Hospital Clínic - Universitat de Barcelona, Barcelona, Spain; 2 Centre for Social Science and Global Health, University of Amsterdam, Amsterdam, The Netherlands; 3 Papua New Guinea Institute of Medical Research, Madang, Papua New Guinea; 4 Infection & Immunity Division, Walter & Eliza Hall Institute, Parkville, Victoria, Australia; Iranian Institute for Health Sciences Research, ACECR, Iran (Islamic Republic Of)

## Abstract

**Background:**

Appropriate antenatal care (ANC) is key for the health of mother and child. However, in Papua New Guinea (PNG), only a third of women receive any ANC during pregnancy. Drawing on qualitative research, this paper explores the influences on ANC attendance and timing of first visit in the Madang region of Papua New Guinea.

**Methods:**

Data were collected in three sites utilizing several qualitative methods: free-listing and sorting of terms and definitions, focus group discussions, in-depth interviews, observation in health care facilities and case studies of pregnant women. Respondents included pregnant women, their relatives, biomedical and traditional health providers, opinion leaders and community members.

**Results:**

Although generally reported to be important, respondents’ understanding of the procedures involved in ANC was limited. Factors influencing attendance fell into three main categories: accessibility, attitudes to ANC, and interpersonal issues. Although women saw accessibility (distance and cost) as a barrier, those who lived close to health facilities and could easily afford ANC also demonstrated poor attendance. Attitudes were shaped by previous experiences of ANC, such as waiting times, quality of care, and perceptions of preventative care and medical interventions during pregnancy. Interpersonal factors included relationships with healthcare providers, pregnancy disclosure, and family conflict. A desire to avoid repeat clinic visits, ideas about the strength of the fetus and parity were particularly relevant to the timing of first ANC visit.

**Conclusions:**

This long-term in-depth study (the first of its kind in Madang, PNG) shows how socio-cultural and economic factors influence ANC attendance. These factors must be addressed to encourage timely ANC visits: interventions could focus on ANC delivery in health facilities, for example, by addressing healthcare staff’s attitudes towards pregnant women.

## Introduction

### Background

Globally, approximately 515,000 women die from pregnancy-related complications each year.[1] In 2010, for Papua New Guinea (PNG), the infant mortality rate (deaths per 1,000 live births) was estimated at 57.[2] As a result of studies’ different methodologies [3] and underreporting of deaths occurring early in pregnancy and in rural areas, the estimates of Maternal Mortality Ratio (MMR) vary considerably: 2008 data from the United Nations Development Programme (UNDP) suggest 250 (maternal deaths per 100,000 live births) whereas 2006 data from the Demographic Health Survey (DHS), indicate a much higher value: 733.[2], [4], [5] Regardless of their limitations, these data nevertheless indicate an unacceptably high burden of maternal and infant mortality in PNG.

In PNG, as elsewhere, the high levels of mother and child mortality have complex underpinnings though maternal anemia is one potentially important factor: more than half of women in PNG suffer anemia during pregnancy (55%). This increases risk of preterm delivery, low birth weight, stillbirth and newborn death.[6] The high MMR in PNG is also linked to the large number of women who deliver at home, without skilled assistance: estimates suggest that only 44% of births are supervised by medical professionals [7].

Antenatal care (ANC) delivered at health facilities is a key strategy to improve mother and child health. The WHO recommends that a range of interventions be delivered as a part of ANC, many of which effectively reduce morbidity and mortality. For example, prevention and treatment of anaemia through iron and folate supplements and malaria prophylaxis.[8] Moreover, the WHO recommendation for “focused” ANC entail a minimum of four health facility visits (with the first occurring during the first trimester), if, following a standard risk assessment, the woman and her fetus are judged to be low risk (Table 1) [9], [10].

**Table 1:** Focused antenatal care (ANC): The four-visit ANC model outlined in WHO clinical guidelines.

Goals				
	First visit8–12 weeks	Second visit24–26 weeks	Third visit32 weeks	Fourth visit36–38 weeks
	Confirm pregnancyand EDD,classify women forbasic ANC (fourvisits)or morespecializedcare. Screen,treat and give preventive measures.Develop a birth andemergencyplan. Advise andcounsel.	Assess maternal and fetal well-being. ExcludePIH and anaemia.Give preventivemeasures.Review and modifybirth and emergencyplan.Advise and counsel.	Assess maternaland fetal well-being.Exclude PIH,anaemia, multiplepregnancies.Give preventivemeasures.Review and modifybirth and emergencyplan.Advise and counsel.	Assess maternaland fetal well-being.Exclude PIH, anaemia, multiple pregnancy,malpresentation. Give preventivemeasures. Review and modifybirth and emergencyplan. Advise and counsel.
**Activities**				
**History (ask, check records)**	Assess significant symptoms. Take psychosocial,medical andobstetric history. Confirm pregnancy andcalculate EDD.Classify allwomen (in some cases after test results)	Assess significantsymptoms.Check record for previous complicationsand treatmentsduring thepregnancy. Re-classification if needed	Assess significant symptoms.Check record for previouscomplications and treatmentsduring the pregnancy. Re-classification if needed	Assess significant symptoms. Check record forprevious complications and treatmentsduring the pregnancy. Re-classification if needed
**Examination (look, listen, feel)**	Complete general, andobstetrical examination, BP	Anemia, BP, fetal growth,and movements	Anemia,BP, fetal growth,multiple pregnancy	Anemia, BP, fetal growth and movements,multiple pregnancy, malpresentation
**Screening and tests**	Haemoglobin; Syphilis;HIV; Proteinuria; Blood/Rhgroup*; Bacteriuria*	Bacteriuria*	Bacteriuria*	Bacteriuria*
**Treatments**	Syphilis; ARV if eligible;Treat bacteriuria if indicated*	Antihelminthic**, ARVif eligible Treat bacteriuria if indicated*	ARV if eligible Treat bacteriuria if indicated*	ARV if eligible;If breech, ECVor referralfor ECV Treatbacteriuriaifindicated*
**Preventive measures**	Tetanustoxoid; Iron and folate+	Tetanus toxoid,Iron and folate IPTp ARV	Iron and folate; IPTp ARV	Iron and folate ARV
**Health education, advice, and counselling**	Self-care, alcohol and tobaccouse, nutrition, safesex, rest,sleeping under ITN, birth andemergency plan	Birth and emergency plan,reinforcement of previous advice	Birth and emergency plan, infantfeeding,postpartum/postnatalcare,pregnancy spacing,reinforcement ofprevious advice	Birth and emergencyplan, infant feeding,postpartum/postnatal care, pregnancy spacing, reinforcementof previous advice

[adapted from WHO ANC guidelines http://www.who.int/pmnch/media/publications/aonsectionIII_2.pdf] Acronyms: (EDD = estimated date of delivery; BP = blood pressure; PIH = pregnancy induced hypertension; ARV = antiretroviral drugs for HIV/AIDS; ECV = external cephalic version; IPTp = intermittent preventive treatment for malaria during pregnancy; ITN = insecticide treated bednet).

*Additional intervention for use in referral centres but not recommended as routine for resource-limited settings.

** Should not be given in first trimester, but if first visit occurs after 16 weeks, it can be given at first visit.

+Should also be prescribed as treatment if anaemia is diagnosed.

In spite of these recommendations, ANC attendance varies greatly across low-income countries.[11] Due to the poor raw data and regional heterogeneity, which leave many pregnancies unaccounted for, estimates of ANC coverage in PNG vary: between 79% [4], and 33% [7] of women in PNG receive ANC at least once during their pregnancy. Poor ANC attendance has a substantive health impact: for example, women in low and middle income countries who make fewer ANC visits have an increased risk of perinatal mortality and, in particular, stillbirth, [13] and are up to four times less likely to use skilled assistance at delivery.[14] In PNG, one missed ANC visit results in a two-fold increase in incidence of malaria during pregnancy (MiP). [15].

In PNG, previous quantitative research on ANC has highlighted multiple obstacles to ANC and healthcare facility utilization in general: for example, health care system factors, economic, psychosocial and cultural factors, social support systems and women’s personal experiences of ANC.[16], [17], [18] Qualitative studies provide important setting-specific insights into ANC attendance related to cultural context and beliefs about pregnancy and childbirth, [19], [20], [21] and, to date, two qualitative studies of ANC attendance in PNG have been published. One was conducted in 1985 in the capital, Port Moresby, and a second in Goroka, the main town of the Eastern Highlands Province [22], [23].

In Port Moresby, respondents viewed ANC as important, attending for pragmatic reasons and because they believed that ANC would control or prevent pregnancy-related problems. They also stated a preference for hospital delivery. Timing of first ANC visit was influenced by advice from husbands and other women, previous experiences of the clinic and of pregnancy.[23] However, all interviewed pregnant women attended ANC at PNG’s only tertiary level hospital and none of the women attended ANC late in pregnancy. Furthermore, interviews were conducted in the clinic, which, as the author states, could have discouraged criticism of health staff. In the Goroka study, 20 pregnant w omen highlighted multiple facilitators and barriers to ANC attendance: physical, cultural and health system factors. The attitude of health care workers was a main concern but women were generally satisfied with their care [22].

These studies were conducted in regions that are culturally and economically particular: Port Moresby is highly urban, whereas Goroka is remote. In light of the limitations of the qualitative research to date and the regional variation in MMRs and ANC attendance across PNG, further examination of the factors that influence ANC attendance in other settings across PNG is required.

Drawing on data from a larger project exploring the socio-cultural context of MiP in Papua New Guinea, Ghana, Kenya and Malawi, this article therefore aims to examine the factors that influence ANC attendance in Madang Province in PNG. Given its relevance for MiP prevention and control, understanding ANC attendance was a key objective of this research, [24] and questions about why women attend ANC at all and about the timing of their first ANC visit will be addressed herein.

### Setting

PNG has an extremely diverse population of 6.5 million: 850 indigenous languages and thousands of disparate communities. Currently, healthcare is provided at Aid Posts, Health Sub-Centers, Health Centers, District Hospitals and Provincial Public Hospitals, in ascending order of size and resources. Although some are government facilities, approximately 45% (and 49% in rural areas) are church-run.[25] Health care workers include doctors, Health Extension Officers, nursing officers, Community Health Workers, and Aid Post orderlies.[25], [26] In Madang Province, in 2008, there were two doctors at the main hospital (Modilon), 20 HEOs, 106 nursing officers, 191 CHWs at health centers and 124 staff at aid posts.[26] In addition, many villages have village birth attendants (VBAs): trained by health center staff, or retired nurses, they aid with home births. Such assistance is a fairly new concept in PNG where women traditionally have given birth alone in the bush unaided.[27] There is no government policy regarding a standard fee for ANC though most healthcare facilities charge for services [28].

Data were collected in the Sumkar and Madang Districts, in the coastal Madang Province from February 2010 to January 2011. The capital, Madang (pop. 36,000), is a port city located on the northeastern coast of PNG. This study was focused around three areas, each surrounding a health facility: Modilon Hospital in Madang town, Yagum Health Center, and Mugil Health Center on the north coast highway. The main language groups in the Yagum and Mugil areas are Amele and Bargam, respectively. In Madang town, participants came from many different language groups due to the high number of migrants. Modilon is government-run, whereas Yagum and Mugil are part of Lutheran and Catholic missions, respectively. In Madang Province, there is reportedly approximately 63% ANC coverage but only 39% of deliveries are supervised.[25] Nurses conducted ANC visits and the one obstetrician in Madang, at Modilon hospital, attended to complicated deliveries.

## Methods

### Study Procedures

The data collection was conducted by a Barcelona-based social scientist in collaboration with local fieldworkers, fluent in the local language and with social science research experience. Data collection methods included focus group discussions, in-depth interviews, observations in health care facilities and case studies of pregnant women.

Direct observations were carried out in the waiting areas and in examination rooms during ANC clinic hours at the three health care facilities on multiple occasions during the initial phase of data collection to gather contextual data and observe patient-healthcare provider interactions. These facilities were selected for their proximities to Madang town: Modilon in Madang town, Yagum clinic a few kilometers out of town and the site of the PNG Institute for Medical Research (IMR), and Mugil clinic about 60 kilometers along the North Coast road out of Madang.

Nine focus group discussions (FGD) were held with groups of women or men in the communities, ranging from three to ten participants. A total of 94 in-depth interviews lasting from 45 minutes to 90 minutes were conducted: 52 with pregnant women, seven with women who had infants under one year of age (women with babies), 16 with relatives of pregnant women including husbands, parents, and siblings, 12 with community leaders, and seven with health care providers (Table 2).

**Table 2:** Study Respondents and Data Collection Tools.

	Data Collection tool			
Type of respondent	Free listingand sorting	IDI	FGD	Case studies
Community members (women)	8		30	
Community members (men)	5		14	
Pregnant woman (PW)	16	52		27
Women with Babies (WB)		7		
Relatives of pregnant women		16		
Community Leaders	1	12		
Health Care Providers (HCP)	7	7		
**Total**	**45**	**94**	**44**	**27**

To gather more in-depth information throughout pregnancy and post-partum, 27 case studies were carried out. Case study women were visited on a monthly basis at their homes to discuss their pregnancies and experiences with and knowledge of ANC. Their husbands and other relatives were interviewed where possible.

### Participants and Recruitment

Participants were recruited through a combination of random and purposive sampling. Initially, during on-site visits at the three health care facilities all pregnant women present at antenatal clinic were invited to participate in an interview. Additional participants were recruited via a snowballing technique in various villages so as to reach pregnant women who may not have attended ANC or who otherwise would not have been captured in the initial phase. Finally, purposive sampling was used to ensure participants of varying ages, parity, marital status, and gestational ages were interviewed. Purposive sampling was also used to capture health care providers involved in ANC at the three healthcare facilities and community leaders identified in the surrounding villages. (Tables 3 and 4) Case study women were selected based on gestational age (under 7 months), willingness to participate, and accessibility by the research team. An attempt was made to recruit an equal proportion of women from different age groups so as to ensure a diverse set of experiences with pregnancy (Table 5).

**Table 3:** Age and Parity of Pregnant Women and Women with Babies.

	Pregnant Women	Women with babies
**Age**		
≤20	10	0
21 to 30	26	2
30 to 39	14	4
≥40	2	1
**Number of living children**		
0 (1st preg)	10	0
1	14	1
2	9	1
3	10	1
4	3	0
5	3	1
≥6	3	3*
		*one woman had 12 children

**Table 4:** Respondent characteristics.

	Opinion Leaders	HCPs	Relatives	PW	WB
**Area**					
Town	2	3	3	17	0
Yagum	4	3	5	16	5
Mugil	6	1	8	19	2
**Sex**					
Male	6	0	9	0	0
Female	6	7	4	52	7
**Type (OL)**					
Religious leader	4				
Community Leader	3				
Elder	3				
Other	2				
**Type (relative)**					
husband			9		
parent			5		
other			2		

**Table 5:** Case Study Characteristics.

Case Study number	Numbermonthspregnant at initiation	Age	Married	Community	Number of livingchildren(currentpregnancyexcluded)	Relativesinterviewed
**CS4**	4	24	Y	Town	0	
**CS10**	6	24	Y	Town	3	husband
**CS12**	6	29	Y	Town	2	
**CS23**	5	16	N	Town	0	
**CS24**	7	19	Y	Town	1	
**CS26**	6	19	Y	Town	0	
**CS3**	5	27	Y	Yagum	1	husband
**CS7**	5	25	Y	Yagum	2	husband
**CS11**	6	21	N	Yagum	0	father
**CS15**	6	21	Y	Yagum	0	
**CS18**	6	24	Y	Yagum	1	mother
**CS19**	5	23	Y	Yagum	1	husband
**CS20**	7	17	N	Yagum	0	
**CS21**	6	40	Y	Yagum	6	
**CS22**	5	20	Y	Yagum	0	
**CS25**	7	42	Y	Yagum	4	
**CS27**	6	17	N	Yagum	0	
**CS1**	8	35	Y	Mugil	5	husband
**CS2**	5	28	Y	Mugil	3	husband, sister, mother, aunt
**CS5**	5	31	Y	Mugil	2	husband, sister, mother, aunt
**CS6**	6	42	Y	Mugil	8	husband
**CS8**	5	21	Y	Mugil	2	
**CS9**	5	27	Y	Mugil	4	
**CS13**	6	25	Y	Mugil	0	mother
**CS14**	6	20	Y	Mugil	1	
**CS16**	5	21	Y	Mugil	1	
**CS17**	5	27	Y	Mugil	1	mother

All interviews were carried out in English or Tok Pisin (the lingua franca in PNG) by (EA, AA, AA). In one case, a village elder that did not speak Tok Pisin was provided with a translator from her village.

Data collection was carried out using an iterative approach such that the research questions and interview question topics evolved as themes emerged from the data and as saturation was reached on a given subject area. Topics addressed included problems during pregnancy; care seeking behavior during pregnancy; relationships with family members and healthcare providers; and knowledge or experience with malaria, specifically MiP.

### Data Analysis

Group discussions and interviews were transcribed and translated into English when necessary. Free listing and sorting, observations and focus group discussions served to provide initial information regarding the topic area and target population. An initial codebook was developed using established categories based on the original research questions and was revised as themes emerged from the data and through research team meetings where the codebook was discussed and refined (EA and RP). The codebook was flexible and codes were reassessed during data collection and revised according to the emergence of novel themes. Likewise, as major trends and crosscutting themes emerged from the data, these were further investigated in the field. Using the computer software Atlas.ti 6 (Scientific Software, Berlin, Germany), interviews and case study notes were coded and analyzed using the grounded theory approach whereby categories, themes and patterns emerge from the data.[29] In a second phase, data associated with the codes relevant to ANC attendance were extracted and organized by sub-themes so as to draw out key findings. A number of techniques were employed to improve reliability and achieve a comprehensive understanding of the findings: data from a variety of participants and different sources (focus groups, interviews and observations) were triangulated; three researchers carried out data collection to reduce individual bias; and case studies allowed for rapport-building and multiple interviews with a single respondent as well as verification of information in health booklets and through family member interviews.

### Ethical Considerations

This study was reviewed and approved by the Papua New Guinea Medical Research Advisory Committee (MRAC No. 09.01) and PNG Institute of Medical Research Internal Review Board (IRB No. 0905), the IRB of Hospital Clinic Barcelona and local procedures and requirements were followed. Verbal informed consent was recorded for all participants before the start of all interviews. Participation in the study was voluntary and a number of chances were given to participants to refuse interviews and they were informed of their right to not answer all the questions. If a participant agreed to be interviewed but appeared uncomfortable or unwilling to answer questions the interview was ended early.

## Results

### Factors Affecting ANC Attendance

Factors encouraging or discouraging ANC attendance fell into four broad categories: accessibility of ANC, attitudes toward ANC, knowledge of ANC, and interpersonal factors.

#### Accessibility of ANC

A number of accessibility-related factors affects whether women attended ANC clinic (Table 6).

**Table 6:** Accessibility of ANC Quotations.

Accessibility of ANC		
Quotation	Participant	Theme
*There were cars but they were all full. I waited for a while but it was getting late so I started to worry how I would get back afterwards, so I just decided not to go to clinic.*	IDI: pregnant woman, 19, 1^st^pregnancy	*Transportation*
*I asked the women, ‘Why can’t you go to Bunabun, because Bunabun health center is just close to where you live?’ They said, ‘Sister, in the morning all the vehicles are coming this way down to Madang town so it’s easier for us to come this way rather than to go in the other direction.’ That is the reason they give all the time. The vehicles are coming this way so it’s easier to come here. Though they live down by Bunabup they come here.*	IDI: health care provider (Sister in charge, head nurse), Mugil clinic	*Transportation*
***Interviewer: Where would you want to deliver, hospital or village?*** * Respondent: It would be hard to find transport if it’s at night. If it’s in the morning or daylight then it would be easier for me to come to deliver in the hospital.*	IDI: pregnant woman, 26, 1^st^ pregnancy	*Transportation*
***I: Why did your husband bring you here? R: Because I didn’t come [to the ANC clinic] in the first place, he was getting a bit impatient because I was having pains down here [points to abdomen]. I’ve got two kids already one after the other. Yes so this is mythird one now. Because I’ve beenexperiencing these pains and he wanted to know what washappening so he quickly brought me down.”***	IDI: pregnant woman, 26, 3 children	*Familial Support*

Pregnant women and women with babies reported that it was not difficult to sell enough betel nuts or other produce to be able to afford the ANC visit fee (generally about 1 Kina (0.37USD)). Even women who had not attended ANC when interviewed sometimes said this barrier would not be difficult to overcome. However, for others, the compound cost of the clinic fee, transportation and the required baby book (2–5 Kina) was significant enough to deter ANC attendance. Furthermore, by attending ANC clinic, women incurred the opportunity cost of missing a day of household or income-generating work (such as selling produce).

In addition to the associated costs, transport availability also influenced ANC attendance. Indeed, trading routes affected the choice of ANC location. For example, the morning traffic flowed towards Madang as people travelled to sell goods at market and some women therefore journeyed south to Mugil Clinic, towards Madang, rather than a closer clinic to the North. In general, however, transport availability influenced place of delivery more than ANC attendance, especially if a woman waited until the final moments of labor before going to the clinic.

Though accessibility was not an issue for pregnant women who lived very close to a clinic (within 5 to 10 minutes walking distance) some did not attend ANC. By contrast, other women went to great lengths to attend ANC, often travelling on foot, while pregnant, for over an hour. This commitment, contrasted with the ease with which other women who did not attend could have walked to clinic or earned enough money to pay the fees, indicates that accessibility was only part of the story and that other factors could be more influential in determining attendance.

#### Attitudes toward ANC

A number of perceived benefits and disadvantages of ANC served as facilitators or barriers to attendance. (Table 7).

**Table 7:** Attitudes toward and Knowledge of ANC quotes.

Quotation	Participant	Theme
*If she is just sick or if she is pregnant she must stillgo to the hospital. This is due to the reason that they must know how the baby is living inside…they must go to the hospital to get rid of this sickness.*	IDI: husband,35,5 children	*Knowledge of* *ANC Services* *Provided*
*If I get lots of treatment [while I’m pregnant] then when it comes to giving birth it will be a normal delivery. I wanted to have a normal delivery therefore I always went to the hospital to get my supply [of medicine] and I make sure I am up to date in taking the medicine. So when going to the hospital for delivery it will be just normal.*	IDI: pregnantwoman,35,5 children	*Delivery*
*I didn’t want to go quickly to the hospital because the medicine too takes a very long time [to finish] that’s why I decided to wait till six to seven months before I go to the hospital to take medicine or that kind of thing. I do not want to drink plenty medicine so I stayed back at home and didn’t* *go that quickly to the hospital.*	Case Study#6, 42,9 children	*Medicine*
*I have to take that [chloroquine] early because I don’t want my baby to get sick you see. And I’ve heard a lot ofproblems of babies born out of malaria mothers becoming very very sick and I said ‘Oh, I don’t want my baby to get malaria so I must get chloroquine every Sunday.’ So I take two tablets every Sunday. The earlier the better. Even at my earlier stage, you know, I make sure I get chloroquine.*	IDI female communityleader,Mugil	*Prevention* *of malaria*
*If you don’t get any medicine to protect your body and your baby against sickness by coming for clinic, this can kill the baby when you have severe malaria.”*	IDI Pregnantwoman, 32,3 children	*Prevention* *of malaria*
***Interviewer: Why didn’t you come for your check-up while you were four or five months pregnant? Respondent: Because I didn’t feel sick or anything while I was pregnant and didn’t want to frequently visit the clinic.***	IDI: pregnantwoman, 29,2 children	*Treatment*

The duration of ANC visits, which could be a full day’s investment, discouraged attendance, particularly for those who lived far away. Long queues and visit times were due to inadequate space, staff, ANC visit days, and inefficient clinical data recording for the numbers of woman in attendance (particularly at Modilon).

Observed ANC visits at all sites consisted of roughly the same lengthy process. Clinics had separate days for enrolment and subsequent visits. Enrolment days generally brought in fewer women, whereas, on revisit days, there tended to be long queues. There were no appointments and women were seen on a first-come first serve basis. Women began arriving before the clinics opened and most arrived within the first hour. They either waited in line or were given a number and were seen one-by-one or a few a time depending on the number of examination rooms and the number of available nurses. Mugil Clinic had one examination room, Yagum two and Modilon three.

Although the women at the end of the line had often arrived soon after those at the front, they waited far longer, sometimes only to be turned away and told to come return on a different date because clinic hours were over (generally by one or two o’clock). Observations highlighted how health staff spent a considerable amount of time hand-writing information in baby books and duplicating it for each woman in the clinic logbook. None of the clinics utilized electronic medical record keeping.

Women’s desire to obtain medicine often led them to attend ANC. Taking medicine in general was seen as important for the health of the mother and baby, and for an easy delivery, without too much pain. However, *not* wanting to take medicine was also a reason for non-attendance and some women reported side effects such as nausea, vomiting, and light-headedness. Women spoke of both “blood medicine” (iron tablets) and chloroquine, which they were given (if available) at the end of ANC visits. Women and their husbands described the medicine provided only in terms of dosing, often with no explanation of its purpose. Although some respondents took neither medicine, others took one but not the other, and some took both. Indeed, not all women spoke specifically of “chloroquine” and “blood medicine” but referred more generally to “medicine.”

#### Knowledge of ANC services

Respondents’ limited understanding of the specific ANC interventions decreased the importance that they placed on attendance. (Table 7).

Antenatal care was often perceived as important for diagnosing (but not necessarily preventing) sickness. Decisions about ANC were closely linked to desires of safe delivery and to decisions about place of delivery. Some women attended ANC to ensure that they could deliver at the clinic without being chastised for not having attended ANC. Moreover, to deliver at a clinic, women often needed to bring their baby book, which was purchased on their first ANC visit. Others, who planned on delivering at home, used a single ANC late in pregnancy to check that their baby was positioned correctly and generally healthy and to ensure that there would be no delivery complications. Whether a woman planned to deliver at the clinic or at home, ANC was a way to be assured of a smooth delivery.

Malaria was not always seen as a particular threat to pregnant women and no respondent mentioned that first pregnancies presented a higher malaria risk. Although some respondents mentioned prevention of malaria and/or getting chloroquine as a reason for attending ANC, this was not the majority. Often those who knew that malaria was dangerous in pregnancy had witnessed a family member’s illness or had heard an explanation about the importance of taking chloroquine from a relative. Some said that nurses told them about malaria, though others were unclear about the reasons for taking the medicine provided. Others saw attending clinic as important for treatment but not prevention, explaining that they only started attending ANC if they felt sick.

#### Interpersonal factors

Various interpersonal factors influenced ANC attendance (Table 8). These included: familial support; relationship with health care providers; disclosure; and spite.

**Table 8:** Interpersonal Factors Quotations.

Quotation	Participant	Theme
*But now that she is pregnant she is attending antenatal clinic every now and then without the man’s support. Looks like the man never cared about the girl and the baby… I told my daughter, “I am here for you. I will look after you up till the time you want to deliver and I will take you to the hospital.” … It is a must that she goes to the hospital. Like I mentioned before, with childbirth it often occurs that the child is dead and the mother is alive, or vice versa…So I take her to the clinic myself.*	IDI: Fatherof Case Study	*Familial* *Support*
***Interviewer: Do you live with your husband or with your parents?***Respondent: *I am living with my husband and his family.* ***Interviewer: Did your mum come with you to the hospital?*** *Respondent: I have to come down all the way from where I lived, collected my mum and she accompanied me to the hospital. My husband’s family never bothered to take me or accompany me to the hospital so my mum sent word for me to come so she can take me to the hospital.*	IDI: Pregnantwoman, 19,1st pregnancy	*Familial* *Support*
*…if some husband are smart enough they can find transport to bring you to the hospital. But some husbands are slack and they are always full of resentment and jealousy and they forget about you. I was living at my husband’s village and he was mistreating me so I went to go back and stay at my village and give birth there.*	IDI: Pregnantwoman, 45,5 children	*Familial Support*
*When they come to the clinic, we usually ask the mothers: ‘Are you ok?’ and the majority just say, ‘I’m ok.’ Most don’t have the courage to tell us if they have a problem. There was* *one case last week. She came and then I could see that she was not happy and also she had some discharge but she didn’t tell me. When I asked her how she was she just said, ‘I’m ok.’ I told her to lie on the bed and when I tried to get the fundal height, I saw that her panty was very wet with discharge. So I asked about it and she said that she has been experiencing this. So some are not well but they don’t admit it.*	IDI: Nurse,Modilon ANC	*Relationship* *with* *Health* *Care* *Providers*
*Yes, I sometimes I would go and sometimes not as I’ll be scared of the sisters because I haven’t attended clinic. If I go and some kind of problems arise say, they’d get cross at me so I’d be scared to go for fear that they might say that ‘you haven’t being coming for clinics, why is that you don’t come for clinic and now you’re coming because you have this problem!?’*	IDI: Pregnantwoman, 31years old, 5children	*Relationship* *with* *Health* *Care* *Providers*
*I think we can get ashamed in front of the Sisters at the hospital if we aren’t they were not consistent with their attendance.*	IDI: womanwith baby,40, 12 children	*Relationship* *with* *Health* *Care Providers*
*I got pregnant when my first baby was still too small and that* *made me feel a little reluctant to go to the clinic. I was a little ashamed of myself.*	IDI: womanwith baby,22, 2 children	*Disclosure*
*Some of the people they did not plan to get married, they are just friends and laze around together and later they find out that the woman is pregnant. The majority of these women do not go to the clinic because they did it secretly and now they are pregnant. They feel shy to go to the clinic because the other mothers who are pregnant have husbands and they also go to the clinic. You know the mothers, while sitting down and waiting for the clinic they will start to make comments, ‘That woman used to roam around and show off now she is here at the clinic.’ These are some of the comments that people say.*	IDI: womanwith baby,25, 2 children	*Disclosure*
***Interviewer: Why were you feeling shy to go for clinic?*** *Respondent: I was shy because if I go the mothers might talk about me; that this girl is too young or that kind of thing they’d say to me thus, I was feeling shy.*	IDI: pregnantwoman, 19,first pregnancy	*Disclosure*
*I didn’t see my period for a month so I went to stay with my husband… I was ashamed of telling them [my parents]…But he wasn’t happy because he wanted to get another wife. He brought me to his house but then he went and brought another woman to the house and so I ran away. I went to stay at my parent’s house but my father removed me from the house… I was pregnant already so they told me to go back and live with him at his house… They said that they wanted me to stay with my husband and they didn’t want me to stay with them that will only result in me having a fatherless child. So I came to live with my cousin but she gossiped about me and her husband removed me from the house… I was living with her for three weeks now and she hasn’t been good to me…So now I am staying with some Sepiks [ethnic group]… If it is a girl I would give it to the Sepiks because they said that they wanted a daughter… I will deliver the baby and I will give it whoever who wants a child and I will go back by myself… My sister told me that I should go back to the village* *with the child. However I said that I couldn’t go back because my parents will get angry and they would not want my child.* When CS23 gave birth, she stayed in Madang to breastfeed for two weeks, left her baby with the woman she was staying with and returned home to her parents.	Case study 23 is fromManam Island.She is nowliving in Madang.She comesfrom a family of six andshe is thethird born child inthe family. At the time ofdatacollection shewas 16 years oldand was sevenmonths pregnant.	*Disclosure*
***Interviewer: Why didn’t you tell your family that you were pregnant?*** *Respondent: I didn’t want people to talk a lot [about me] so I didn’t tell them.*	IDI: pregnantwoman, 29,2 children	*Disclosure*
*All sorts of problems can arise [during pregnancy and delivery] because the parents do not agree with you marrying a certain man but you disobey to go marry the guy anyways. Later you face the consequence. If I have a miscarriage I will say ‘My parents must have cast a curse on me and that’s why* *I miscarriage.’ So then I must go and apologize to my parents so that they can reunite with me. We will mix gorgor [a ginger root plant] with water and wash with it and drink the water. So then the foetus will grow mature inside and you will be able to give birth well.*	IDI: womanwith baby, 26,2 children	*Disclosure*
*For [my second baby] I did not go down to the hospital quickly because there was some conflict between me and my husband and I have cursed myself that I will die when giving birth. After the curse, the baby turned itself upside down. The nurses then told my father in-law: ‘the baby is sleeping upside down so you have to go and talk to her to at least solve her problem.’ So he came home in the afternoon and he asked* *me about the problem and I lied to him, saying that I am just fine. I did not want to talk to him because I had made up my mind that I was going to die.*	Case Study#7, 25,2 children	*Spite*
*I was offended and feeling depressed so I made up my mind not to go to the clinic for check-up but to just stay in the village. I promised not to go for check-up.*	IDI: pregnantwoman, 45,5 children	*Spite*
*They don’t want to come to the health center. They want to deliver at home because they are not happy with their husbands. They said enough ‘three, four, children is enough.’ But you know the husbands’ mentality; they want to have another one and another one. That’s why the wives are really fed up. We had one last week in here [for delivery]. She had never attended any antenatal clinic and so when she came I asked, ‘Did you come for antenatal clinic?’ and she said ‘No.’ I said ‘why?’ She said ‘I’m fed up with my husband. Seven children is enough. I don’t want number eight but now I have number eight so let me just stay like this and then you know I die. Let me deliver at home and let me die.’ Sometimes they are angry with their husbands, and they do that.”*	IDI: Sister inCharge (head nurse),Mugil clinic	*Spite*

Encouragement and support from husbands, other women with pregnancy experience, parents or in-laws positively influenced ANC attendance. A husband’s support entailed providing his wife with the funds to attend, taking care of other children, cooking food in her absence, accompanying his wife to clinic or encouraging her to attend. In-laws also played a supportive or prohibitive role: for example, accompanying a woman to clinic or, on the contrary, being strict about housework or income, making it harder for her to miss a day of work to attend ANC.

A few pregnant women and women with babies spoke positively about living with their own families during pregnancy in contrast to staying with their in-laws. One option entailed moving from their village of residence to stay with a family member who lived closer to the clinic where women would stay until they delivered, or until the baby had received all immunizations. In these cases, husbands and other children might join the expectant mother, with the children being enrolled into the closest school.

Poor relationships with health care providers or negative experiences at the clinic deterred women from attending clinic (or from attending more than once or twice).

When asked about how they communicated with pregnant women regarding their problems, nurses said that the women would not talk to them about such problems. Women however reported that nurses did not ask, or that they did not feel comfortable talking with the nurses. Lack of privacy may have contributed to this discomfort as most of the examination rooms were within earshot of other women who were waiting their turn, often separated only by a thin curtain. Moreover, there were instances of basic questioning outside the examination room in front of all the waiting women.

In general, observations during ANC visits revealed that generally there was little verbal communication between nurses and pregnant women. Although no severe verbal abuse was observed, nurses were generally curt rather than warm. Pregnant women were asked, “Are you alright?” to which they would generally respond, “Yeah, I’m fine,” without further follow up. At most, there was occasional inquiry into the woman’s experiences of various specific symptoms (including swollen feet and stomach pains), using a simple checklist of potential problems, though this was not consistently observed across all visits.

Women reported being afraid of nurses’ reprimands. They said that nurses were unpleasant and, at times, would yell at them. Nurses on the other hand described how they wanting to help the women, but talked of being overworked and under-staffed. They also expressed frustration with the women who attended ANC late in pregnancy or who did not heed their advice.

Women also reported that sometimes nurses scolded them for inadequate birth spacing. Appropriate birth spacing was widely viewed as important within the community: respondents often reprimanded themselves or others for not having “good spacing,” which was described as not conceiving until the previous child could walk and take care of him/herself to a certain extent. A recurrent discourse surrounding spacing referred to the past as a time “good spacing” in contrast to the present day. The blame was often placed on husbands for not leaving their wives alone, and alcohol was sometimes cited as a cause of this.

Fear of reprimand could dissuade women from attending ANC. Opinion leaders described a cycle whereby women did not attend ANC in early pregnancy and then would be afraid to attend later because of the nurses’ chastisements. Clinics reputations regarding “nice” or “harsh” nurses varied and women reported that they therefore might travel further to attend a preferred clinic.

Stigmatized groups were reportedly less likely to attend ANC because to others this was a clear signal of pregnancy. Fear of embarrassment, gossip, nurses’ reprimands or family members learning of the pregnancy could discourage women from attending ANC. Some women feared sorcery that might harm the mother or the child, even from their parents.

Adolescents, single women for whose pregnancy no man acknowledged paternity and women without “good” birth spacing were stigmatized. Although young pregnant women sometimes received support from their mothers or other relatives who brought them to the clinic, in other instances parents were angry with their daughters for getting pregnant. A young woman might leave home for the duration of her pregnancy, give up her baby upon delivery, and then be allowed to return to her family. Notably, even women who were in relationships did not always disclose their pregnancy to family members for fear of gossip.

A number of women spoke about not seeking care as an expression of depression or in response to arguments with their husbands. Not seeking care was sometimes described as a way of punishing husbands for treating women badly. For others, not attending ANC was a form of protest against husbands for unwanted or untimely pregnancies. The ultimate goal of such protests was unclear: whether they intended to miscarry and/or commit passive suicide, whether they were a demonstration of their depression, or whether they were a symbolic expression of their dissatisfaction.

### Timing of First ANC Visit

A variety of factors influenced timing of first ANC visit (Table 9).

**Table 9:** Timing of first ANC Visit.

Quotation	Participant	Theme
*Today I had one [patient] at one and a half months [pregnant] - too early. But she came. You know these new mothers – they are so anxious. So I checked her but I told her, you go stay [at home] until you are four or five months. At four or five months, you come for antenatal clinic.”*	IDI: Headnurse,Mugil clinic	*Perception*
***Interviewer: And when do you think they should come for the first time? What do you think would be best? Respondent: When they miss the period. That would be the best time…That’s the first visit but the next visit date we give them is 8 to 10 weeks later.***	IDI: nursemidwife,Modilon clinic	*Perception*
*Even when I was four months pregnant, I still did not feel I was pregnant, I felt like I did not have a child, you know. That made me not want to go to the hospital for clinic until I was five months. Then I felt the movement of the baby and I thought, ok: I will have to go to the clinic next month.*	Case Study #4,28,3 children	*Strength* *of Fetus*
*After I stayed at home for some time, I felt that the baby moved* *and so I came to the hospital to check on the baby*	IDI: pregnantwoman, 37,4 children	*Strength* *of Fetus*
**Interviewer: Why did you decide to come at three months that time?**Respondent: This is my first time and I was a bit scared. I had never experienced such thing so because it’s my first time I came when I was three months.	IDI: pregnantwoman, 29,3 children	*Previous* *experiences*
*I don’t usually go for clinic at four months but I when was just four months but my belly had grown so huge it was making me worry a lot, so I went for clinic. With the other three boys, I went to start clinic at six or seven months.*	IDI: pregnantwoman 31,4 children	*Previous* *experience*
*But for this baby now I stayed till six months and I went to the clinic. This is because I don’t have money to pay for the PMV to go to and fro. Further still I do not have money to pay for the hospital bills as well as the clinic card so I stayed till six months when I have enough money and I went to the clinic.*	IDI: PregnantWoman, 35,5 children	*Repeat* *visits*

#### Perceptions of appropriate timing

Pregnant women, relatives and opinion leaders generally reported that the best time to start going to ANC was around four or five months. However, women generally reported initiating ANC at six or seven months and some as late as eight months. Women potentially received mixed messages regarding the ideal time to initiate ANC. Three providers said around three or fours months was ideal and might send away women who came earlier, telling them to return after quickening. Other nurses stated that women should attend as soon as they missed menstruation.

#### Strength of the fetus

Health care providers and pregnant women explained ANC initiation in terms of waiting until the baby was strong enough, when the mother could feel it moving and her belly was noticeably larger. Although women recognized pregnancy after one or two missed menstrual periods, carrying to term was considered more probable after quickening, and thus care could be sought and the pregnancy disclosed to extended family members.

On the other hand, fears of one’s baby getting “too big” before delivery as a result of taking medication could discourage early ANC initiation. In such cases, women waited until they could only attend once or twice before delivery.

#### Past experiences

Parity and past pregnancy experiences affected ANC initiation. In general, multiparous women attended ANC later compared to primigravidae. Often nervous during a first pregnancy, women would attend as soon as they knew they were pregnant. Multiparous women felt less inclined to attend early and would sometimes attend only once close to the time of delivery.

Women who had experienced problems during a previous pregnancy – a miscarriage or delivery complications – or had witnessed such problems, were likely to initiate ANC earlier. Likewise, discomfort or problems during a current pregnancy (particularly if they had not previously occurred) could also prompt women to initiated ANC earlier.

#### Multiple visits

The most common explanation for delaying ANC initiation was a desire to avoid multiple clinic visits. Women and health care providers explained that the later in a pregnancy a woman attends ANC, the fewer visits she has to make before delivering. In early pregnancy (up to 28 weeks) women were scheduled monthly appointments; from 30 to 36 weeks, appointments were every two weeks; and in the final weeks, women attended weekly until delivery. Pregnant women perceived ANC attendance as compulsory once an initial visit had been completed and case study women generally completed these follow-up visits. Multiple visits compounded the undesirable effects attending ANC, described above, such as (direct and opportunity) costs or having to take medicine.

Timing of first ANC visit could also be determined by the time it took women to save the money to meet the transport costs and clinic charges. Distance from a clinic also was particularly important for timing of ANC initiation (and thus the number of total visits), rather than whether a woman attended ANC at all. However, pregnant women, relatives and health care providers also described first visit delays as due to “laziness.”

## Discussion

This qualitative study describes the multitude of sociocultural factors affecting ANC attendance in Madang, Papua New Guinea. Although attending ANC was generally viewed as important, a wide variety of factors encouraged and/or discouraged ANC attendance and affected when women initiated ANC. These factors fell into four categories: accessibility, attitudes, knowledge, and interpersonal factors. There were no notable patterns of views across age, respondent type or clinic.

### Accessibility of ANC

The cost of and distance to health facilities had a limited effect on whether or not a woman attended ANC at all and were more likely to affect the timing of ANC and place of delivery. Another study conducted in Madang also found that living closer to a health facility does not necessarily lead people to seek treatment. Furthermore, cost was not a significant factor for choosing a formal health care facility.[28] Some pregnant women included in this study went to significant lengths to attend ANC by, for example, staying with a family member who lived closer to a clinic throughout their pregnancy, sometimes taking husbands and other children with them. As has been found in similar studies (in PNG and in other low-income settings), [22] transport cost and availability influenced some women’s decisions regarding when to attend ANC.

Women choose health facilities based on advice from husbands or others, or because of perceptions regarding better quality and effectiveness.[23], [28] In general, women preferred health centers over the hospital in Madang.[23], [28] Some moved to live closer to a health facility because it meant staying with their own family, where some felt they would receive better care than if they lived with their husband’s family.

Familial support or lack thereof served as a facilitator or barrier to ANC attendance. Some family members helped women travel to the clinic, provided financial assistance, or helped with the housework. Indeed, husbands’ support has previously been found to be an important facilitator for ANC attendance in PNG [22], [23].

### Attitudes toward ANC

Previous negative experience of ANC could discourage women from attending. As in other settings, long waiting times (after already long journeys), [22], [30], [31], [32], [33], [34] unannounced clinic closures, [22] and lack of medications, [22] were common complaints and led some women to make little effort to attend ANC. An ANC visit sometimes required a whole day. This, along with associated opportunity costs (household or income-generating labor), could discourage women from attending at all, or reduce their number of visits. Therefore women’s heavy workload and limited economic opportunities influence on ANC attendance.[19], [35] Wage-earning women in the more urban setting of Port Moresby sometimes found it difficult to take time off work to attend ANC.[23] The findings indicate that in Madang women did not see village birth attendants as a preferable ANC option as has been found in other settings [19], [32].

Obtaining medicine encouraged and discouraged ANC attendance. Some women spoke of the importance of receiving malaria medication and iron tablets for their own health and that of their baby (ensuring a safe delivery).[22] Women in Port Moresby also attended ANC because they wanted to receive *the correct* amount of medicine.[23] Others however did not like taking the medication usually because of the side effects that they experienced. Women generally reported that by attending ANC and being offered medicine, there were obliged to take it. This discouraged women who didn’t like medicine from attending.

### Knowledge of ANC

Pregnant women and their family members considered ANC to be important. However, most respondents had a limited understanding of specific ANC interventions and saw ensuring a safe delivery as the main benefit of attending the clinic. As has been found in qualitative research in PNG and elsewhere, women particularly focused on checking the position of the baby [22], [23], [36].

Perceiving ANC as a place for curative treatment rather than illness prevention sometimes discouraged women from attending. Along with women in Goroka and sub-Saharan Africa, respondents in this study reported attending ANC to discover their sickness.[22], [37], [38] Thus, some women (and their husbands) did not see the value of attending ANC if a woman feels healthy and they thought there was nothing wrong with the fetus.[30], [31], [32], [34], [39], [40], [41] Other studies have shown this tendency to perceive bio-medicines as being curative rather than preventative.[32], [42] Similarly, perceptions regarding low malaria risk during pregnancy discouraged ANC attendance. Few respondents expressed a need to take chloroquine during pregnancy and malaria was not seen as a particular threat to pregnant women.[43] No respondent mentioned that first pregnancies present a higher malaria risk.

### Interpersonal Factors

A woman’s perceptions of health care providers could affect whether she attended ANC or which facility she attended. Women sometimes described feeling disrespected by health staff, whereas the nurses reported frustration when women did not heed their advice. Research commonly highlights that women complain of abuse, neglect, mistreatment or intimidation at health centers, while health workers describe women as unhelpful, [20], [44], [45], [46] and that reprimands from health staff can discourage attendance [30], [33], [47], [48], [49], [50], [51], [52], [53], [54].

Previous observations in PNG have confirmed that there is limited communication between pregnant women and health care providers and that they have little time for relationship development: women didn’t ask questions and health staff were often abrupt, condescending and shaming.[22], [23] suggest that this public shaming of women forms part of an attempt to increase compliance, assuming that they will comply to avoid this public shaming. Though this research was conducted in a different region of PNG, it is possible that such rationale could apply in Madang where health care providers also have significant perceived power over their pregnant patients and, as Larsen et al. point out, such attitudes might be expected because these relationships are embedded in a culture where there is a general acceptance of the “directions and advice from a ‘superior’…particularly in relation to public or government services.” [22].

Furthermore, women’s reluctance to ask questions may be shaped by the expectation that one “respect others who are viewed as more knowledgeable, which in this case is expressed through listening, and a desire to avoid being shamed; therefore, the women remain silent.” [22] Women in Madang may also have been hesitant to talk about their health problems due to lack of privacy or may have also considered certain symptoms to be normal and thus not worth reporting.

In Madang, as elsewhere, attending ANC results in pregnancy disclosure to other community members.[55] However, women may not want to disclose a pregnancy for a range of reasons [19], [32], [34], [36], [40], [50], [55], [56], [57], [58], [59] and in Madang women were particularly reticent to disclose a pregnancy if they did not maintain “good spacing” or if no man claimed paternity.[23] Furthermore, as has been found in other contexts, [34], [60], [61], [62] some may want to keep their pregnancy secret to avoid harm from other people or spirits. For many people in PNG, health and illness are linked to spirits, ghosts and sorcerers, although a death may be due to malaria, for example, its deeper cause is commonly explained in terms of sorcery.[63] In some African and Asian settings, fears of jealous contemporaries “cursing” pregnant woman was found to have a detrimental effect on pregnancy disclosure [34], [50] but no such fears were identified by respondents in Madang.

Disclosing a pregnancy by attending ANC emerged as a major concern for women who viewed their birth spacing or parity as socially unacceptable. However, many did not have the information or means to prevent unwanted pregnancies. Thus, some women saw their pregnancy as their husbands’ fault and expressed despair, anger and a desire to punish their husbands by hurting themselves or the baby by not attending ANC. Taking one’s own life or that of a child echoes Wardlow’s work on “passenger women” in PNG, which found that women who accepted money for sex sometimes did so in order to “ruin” themselves, to strip their male family members of the opportunity to profit from their bride price, or to shame them, precipitated by incidents of violence, loss or humiliation by male relatives [64].

### Timing of First ANC Visit

The factors influencing whether women attend ANC also impacted on when ANC was initiated. Many respondents spoke of wanting to avoid repeat clinic visits and there was a general perception that once you started attending to clinic, you were compelled to attend all follow-ups. This pressure may come from the previously discussed power-filled relationship between health care providers and pregnant women, though precisely what caused this feeling of obligation was not entirely clear as women were also reprimanded for delaying ANC initiation. This perceived compulsion confirms a similar finding of ANC attendance in Goroka, where all of the women in the study attended regularly after an initial visit. All woman in that study continuing to attend antenatal care once she had started [22].

Other factors influencing ANC initiation echoed findings from studies in other settings. The higher a woman’s parity, the later she would attend the ANC.[22], [32], [40], [48] Some would only attend once, shortly before delivery, to check for any possible complications or to facilitate a clinic birth. Waiting until the baby was strong before attending was similar to other contexts where women waited until they had missed several periods before confirming a pregnancy.[19], [30], [31], [39], [48], [49] Women in Port Moresby also reported minimizing the total number of ANC visits because they disliked the medicine given at clinic, there were other demands on her time or transport was difficult [23].

Broader issues highlighted here that affect ANC attendance and MiP care and require further exploration. Indeed, more social science research is needed specifically on problems during pregnancy, quality of ANC services, and family planning and delivery in the PNG.[65], [66] Moreover, because this and previous studies largely interviewed women who attended ANC at least once, focusing on women who never attend ANC is a priority.

### Strengths and Limitations

The use of qualitative methods enabled an in-depth understanding into the mechanisms influencing why and when women attend ANC clinic in Madang, PNG. Multiple interviews with case study women for the duration of their pregnancies and interviews with their family members particularly aided in uncovering the multidimensional reasoning behind women’s decision-making process. Moreover, as it is the first study of its kind to be conducted in the Madang region, it fills a key gap in the literature.

One main limitation of this study includes the fact that almost all the women interviewed attended ANC. Most of the women interviewed lived near a clinic, a main road, or had moved to be closer to a clinic during pregnancy. It is therefore possible that for those living even further from clinics, distance is a stronger barrier. The use of the snowballing technique in villages did however ensure that the study recruited pregnant women from outside the clinic setting.

Some case study women who had not attended ANC clinic during early phases of research may have felt pressured to attend to avoid telling the researchers that they had not yet gone. This observation bias, or Hawthorne effect, may also have affected nurses’ behaviour during clinic visit observations.

Another limitation of this study is that the initial intent of the research project was to investigate the social and cultural context of malaria during pregnancy. Thus, during data collection less emphasis was placed on facilitators and barriers to ANC. However, this was a salient issue that arose during data analysis.

### Policy Recommendations

A number of policy recommendations can be drawn from the findings of this study.

Clear and consistent messaging regarding ANC could include information about the ideal time to first attend ANC, helping pregnant women and their families know when to attend. Improving knowledge of the services provided at ANC clinic and their purposes would also be beneficial. Messaging could also emphasize the importance of preventative medicine and ensure women and their communities know that attending ANC is not only to ensure ease of delivery but for the overall health of mother and child.

Better information about and provision of family planning could also reduce unwanted pregnancies. Family members could also be empowered to support their women financially, emotionally and physically by helping pay for ANC attendance, encouraging women to attend, and accompanying them or helping with housework in their absence.

Clear and consistent messaging regarding possible complications during pregnancy would include information regarding the possibility of complications during all pregnancies, not just the first, encouraging women of grandmultiparas to attend ANC clinic. Furthermore education about malaria, HIV and other diseases that cause morbidity and mortality amongst pregnant women and infants is key, as is explaining the importance of taking medicine to prevent or treat these illnesses.

Structural changes could also increase ANC attendance. This could include longer ANC hours on more days and rather than sending pregnant women away (either because they attend on the wrong day or too early in her pregnancy), they could always have the opportunity to be seen by a health care provider. This would require resources for more ANC nurses to be trained and hired as well as increased space for examination rooms. Increased privacy of examination rooms could also encourage women to disclose problems they are facing.

Although electronic medical record keeping was not observed in any of the clinics, the introduction of this technology could reduce the time that is wasted transcribing information to health books and clinic logs and could improve continuity of care.

Current best practice guidance places an emphasis on routine screening, testing, and health education topics, rather than on individual concerns and circumstances.[67] Increased concentration on individual concerns and an understanding of a woman’s circumstances would encourage women to seek care. Furthermore, kindness from health care providers combined with concrete solutions and support, rather than counter-productive shaming of high parity women or those with perceived “inadequate” birth spacing could encourage ANC attendance. Although reprimands may work in some cases, they are not an effective method in Madang. Furthermore, decreasing stigma linked to these two issues combined with better education about and access to family planning would be beneficial.

## Conclusion

Overall, ANC was perceived as an important part of ensuring a safe delivery for both mother and child. However a number of factors facilitated and discouraged ANC attendance. These were related to accessibility, attitudes, knowledge, and interpersonal factors. Consistent and empathetic messaging from health care providers, longer ANC hours, increased provision of family planning together with greater information and encouraging relatives to ensure women can attend a health facility could help increase (and quicken) ANC attendance.
